# Polarization-independent narrowband photodetection with plasmon-induced thermoelectric effect in a hexagonal array of Au nanoholes

**DOI:** 10.1515/nanoph-2024-0643

**Published:** 2025-02-17

**Authors:** Sehyeon Kim, San Kim, Jae-Young Kim, Tae-In Jeong, Munki Song, Seungchul Kim

**Affiliations:** Department of Cogno-Mechatronics Engineering, College of Nanoscience and Nanotechnology, 34996 Pusan National University, Busan 46241, Republic of Korea; Department of Optics and Mechatronics Engineering, College of Nanoscience and Nanotechnology, 34996 Pusan National University, Busan, 46241, Republic of Korea

**Keywords:** surface plasmon resonance, narrowband photodetection, photothermoelectric

## Abstract

Photodetectors are crucial for modern technologies such as optical communications, imaging, autonomous vehicles, and machine vision. However, conventional semiconductor-based photodetectors require additional filtering systems due to their broad spectral response, leading to increased costs and complexity. Here, we present a narrow spectral response photodetector using hexagonally arranged plasmonic Au nanohole structures, eliminating the need for optical filters. The device achieves a full-width at half maximum (FWHM) bandwidth of ∼40 nm with a response peak at 760 nm and a linear photocurrent responsivity of 0.95 μA/W. The photothermoelectric effect, induced by the nonradiative decay of plasmonic resonance, converts optical radiation into an electric potential on the Au surface. The hexagonal nanohole design generates polarization-independent photocurrents and allows spectral tuning beyond the cutoff region of silicon photodetectors. This versatile approach enables customizable response characteristics across a broad wavelength range through geometric design, enhancing its potential for diverse applications.

## 1 Introduction

Photodetectors are crucial components in various systems that utilize light as a signal carrier, such as optical communications [1], [2], chemical analysis [3], autonomous vehicles [4], and machine vision [5], [6], [7]. Photodetectors can be classified into broadband and narrowband types based on their spectral response bandwidth, with each type offering distinct advantages depending on the intended application. Narrowband detectors, the focus of this study, are particularly advantageous for detecting a narrow range of wavelengths. This selective detection minimizes noise by avoiding extraneous signals from the surrounding environment that fall outside the target wavelength range, thereby enhancing image resolution and sensing accuracy. Conventional photodetectors, typically based on semiconductor materials such as Si, GaP, and GaN, offer high optical sensitivity, but their spectral response is inherently limited by the bandgap energy of the semiconductor, functioning similarly to a low-pass filter. This limitation complicates the realization of a narrowband spectral response. Consequently, optical filter systems are often combined with semiconductor detectors to operate as narrowband photodetectors [8], [9], [10], but this approach can lead to increased cost, size, complexity, and energy losses due to additional optical interference or scattering [11], [12], [13], [14]. Conventional dichroic bandpass filters, which achieve selective transmission and reflection properties through the repeated deposition of high- and low-refractive index materials on a glass substrate, are particularly challenging to miniaturize to a chip scale or integrate with photodetectors [15], [16]. Therefore, novel approaches that enable chip-scale, narrowband photodetection without reliance on semiconductors are needed.

In this context, numerous studies have reported narrowband photodetectors that selectively absorb specific wavelengths through the deposition of quantum dot (QD) materials on sensor surfaces [16], [17], [18], [19], [20]. However, achieving narrowband sensors with diverse wavelength selectivity requires new synthesis and deposition processes for suitable quantum dot materials. In place of QDs, surface plasmons (SPs) provide an alternative method for selective optical absorption in metals. Metals such as Au and Ag, which exhibit high negative permittivity in the visible or infrared (IR) range, can induce strong collective oscillation of free electrons at the interface between the metal and dielectric surroundings [21], [22]. These oscillations occur at specific wavelengths and can be manipulated by designing the geometrical parameters of the nanostructure. This light–matter interaction converts the energy of incident light into the collective motion of electrons, generating intense, localized near-fields around nanostructures with specific shapes. The local fields generated by SP resonance can enhance the efficiency of optical absorption or nonlinear harmonic generation in nanoscale materials such as QDs [20], [23], [24] and two-dimensional (2D) materials [25], [26], [27]. Interestingly, SPs can directly convert optical energy into electric potential on the metal surface. The near-field generated by irradiated light can decay radiatively, emitting a photon, or nonradiatively, generating hot electron–hole pairs [28], [29]. These high-energy hot electrons undergo relaxation over 100 fs to 1 ps, redistributing their energy as heat to the surrounding materials [30], [31]. This process creates localized temperature increments within the metal film, converting light energy into electrical potential through the localized Seebeck effect. Unlike traditional semiconductor-based photodetectors, this photodetection mechanism is not constrained by the material’s bandgap energy and requires no additional bias voltage [32]. Furthermore, by adjusting the geometric parameters of the plasmonic structure, the resonant wavelength range can be precisely tuned across a broad region of the infrared spectrum.

In this study, we demonstrate a narrowband photodetector by exploiting a hexagonal array of Au nanoholes to induce a narrowband plasmonic photothermoelectric (PTE) effect. The hexagonal array of nanoholes exhibits nearly equivalent PTE effects for any polarization state of the irradiated light. The designed plasmonic structure shows resonant field enhancement at a wavelength of 760 nm, while the maximum optical transmission peak occurs at 770 nm due to a discrepancy between surface and longitudinal plasmonic resonances. The plasmonic PTE device demonstrates a linear photocurrent responsivity of approximately 0.95 μA/W when irradiated with a continuous wavelength laser at 770 nm under zero-bias voltage, with a narrow full-width at half maximum (FWHM) spectral response of ∼40 nm. Additionally, unlike a square array of plasmonic nanoholes, the hexagonal array enables stronger plasmonic interactions due to the simultaneous excitation of multidirectional plasmon modes arising from the hexagonal arrangement [33]. This configuration results in higher field enhancement and more efficient photocurrent generation. Experimental results and numerical simulations indicate that local heat generation in the hexagonal array is independent of light polarization, reducing polarization-dependent energy losses and the need for calibration in photodetection. We also numerically calculated the tunability of the resonant wavelength across a range from the visible to the near-infrared spectrum (specifically from 650 nm to 1,150 nm) by adjusting the pitch and hole diameter of the Au nanohole array. This ultra-compact, chip-scale PTE-based narrowband photodetector holds potential for advanced applications in sensing, imaging, and quantum communications.

## 2 Results and discussion

### 2.1 Principle of plasmonic PTE effect and its characteristics

**Figure 1:**
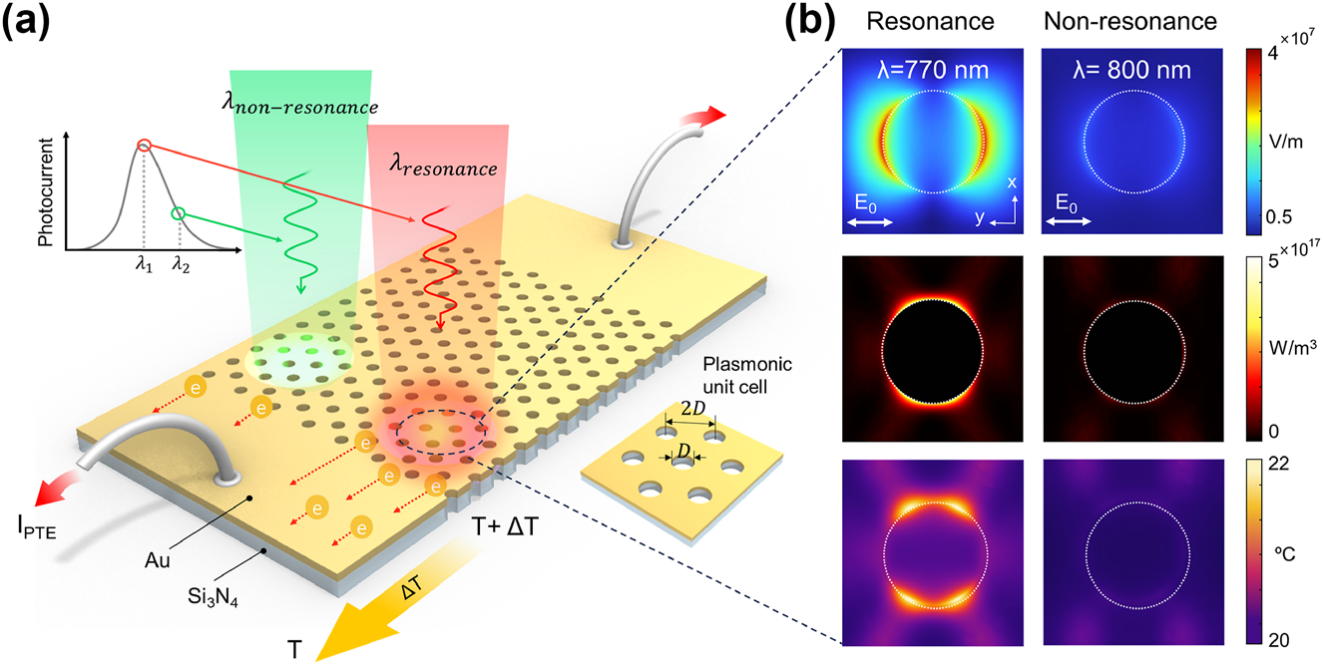
Concept of narrowband plasmonic PTE photodetection. (a) Schematic illustration of the narrowband plasmonic PTE photodetector. The plasmonic PTE effect is strongly induced by an incident laser beam with a center wavelength matching the SP resonance condition (shown in red), while a weak PTE effect is observed under the nonresonant SP condition (shown in green). The local near-field generated by the SP resonance causes temperature increments around the nanoholes. Free electrons in the Au film move from the hot region to the cold region via the Seebeck effect. (b) Calculated intensity distribution (top), heat source density (middle), and heat distribution (bottom) at the Au surface. Wavelengths of 770 nm and 800 nm were selected for surface plasmon resonance and non-resonance conditions, respectively, with the power of the incident light set to 10 mW in both cases.

Figure 1a shows a schematic of the narrowband photodetector utilizing the plasmonic thermoelectric effect. The photodetector is composed of a hexagonal array of Au nanohole structures fabricated on a thin Au/Si_3_N_4_ substrate. This Au film, perforated with nanoscale periodic holes as illustrated in the figure, supports extraordinary optical transmission (EOT), where the transmission efficiency through the subwavelength apertures is strongly enhanced when SPs are resonantly excited by the irradiated light [34], [35], [36]. Thus, measuring EOT is useful for experimentally evaluating the SP resonant wavelength. The excited SPs either transfer their energy through a radiative decay process, such as EOT, or lose energy through nonradiative decay, generating hot-electron pairs, which are converted into heat within the metal structure during their relaxation process [30]. This heat leads to a temperature difference between the plasmonically excited holes and other regions, inducing a photocurrent in the device via the Seebeck effect. The thermoelectric voltage (Δ*U*) generated by the Seebeck effect is expressed as Δ*U* = −*S*Δ*T*, where *S* is the Seebeck coefficient of the material and Δ*T* is the temperature gradient [32]. This equation shows that higher photocurrent can be induced when a stronger temperature gradient is formed due to the intense field enhancement from the SP resonance (colored in red). On the other hand, when non-resonant light (colored in green) is irradiated on the same plasmonic structure, the SP resonance is weakly induced, leading to low photocurrent due to an insignificant temperature gradient. The amount of photocurrent corresponds to the resonant characteristics of SPs, enabling a narrowband photodetector that generates current only near the SP resonance wavelength.

The analysis of the local heat source distribution induced by SPs was performed to investigate the plasmonic photocurrent generation process. The finite element method (FEM) was used to calculate the local electromagnetic fields and the local heat source distribution around the Au nanohole. The hexagonal array of nanoholes was modeled as a rectangular unit cell. A perfectly matched layer (PML) was applied to the planes orthogonal to the optic axis of the unit cell, and periodic boundary conditions were applied to the other boundary planes. To align with the experimental conditions, a plane wave excitation with an average power of 10 mW was applied in these calculations. Figure 1b shows the simulation results for the electric field (E-field) (top panel), heat source density (middle panel), and temperature distribution (bottom panel) generated by the laser irradiation on the hexagonal Au nanohole array. The initial temperature of the nanohole was set to 20 °C, near room temperature. We designed the hexagonal array of Au nanoholes on an Si_3_N_4_ substrate to achieve maximum optical transmission at a wavelength of 770 nm (top-left panel), displaying a strong electromagnetic field enhancement at the edge of the nanohole. In contrast, laser excitation at a wavelength of 800 nm, only 30 nm from the plasmonic resonant wavelength, did not produce any significant field enhancement. This discrepancy in electric field enhancement leads to a different local heat source within the nanohole.

When light at the resonant wavelength is incident on the nanohole array, a strong electric field (top-left panel) is generated along the polarization direction of the incident light at the aperture edges. The heat source density, reflecting power dissipation from the excited local electric field, is localized along the aperture edges and distributed perpendicularly to the local electric field. This suggests that the excited SPs experience nonradiative energy loss during their collective oscillation along the arc of the nanohole. In the resonant mode, the temperature was predicted to increase by up to 2 °C from the initial temperature of 20 °C (bottom-left panel). In contrast, in the non-resonant mode, due to the weaker E-field compared to the resonant mode, no distinct heat source was generated, and the temperature distribution remained at the initial 20 °C (bottom-right panel). These simulation results clearly demonstrate that SP resonance significantly affects photocurrent generation and that the formation of localized heat sources during the nonradiative decay process of SPs leads to a noticeable temperature increase along the arc of the nanohole.

**Figure 2:**
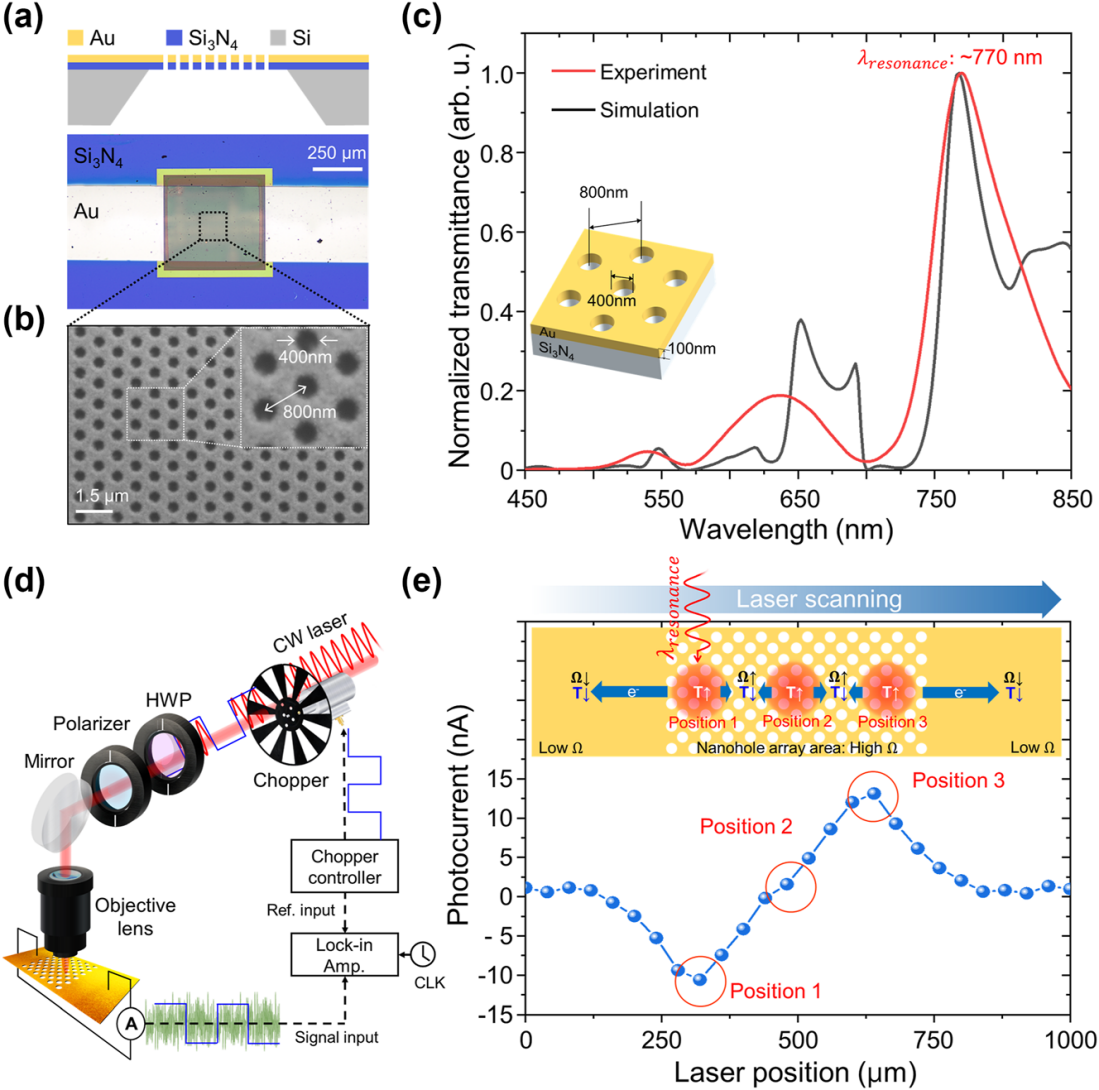
The experimental setup and optical/PTE characteristics of the Au nanohole array. (a) Schematic image and optical microscope image of the PTE device, showing an Au nanohole array deposited on a Si_3_N_4_ membrane. The fabricated nanohole array has a diameter of 400 nm, a pitch of 800 nm, and a thickness of 100 nm on a 200 nm-thick Si_3_N_4_ membrane. The nanohole array size is approximately 500 μm × 500 μm. (b) SEM image of the Au nanohole array, arranged in a hexagonal pattern with a hole diameter of 400 nm and a pitch distance of 800 nm. The Au film thickness is 100 nm. (c) Theoretical (black line) and experimental (red line) transmittance spectra through the PTE device. (d) Schematic illustration of the experimental PTE setup: the external cavity diode laser (ECDL) is modulated by a chopper synchronized with a lock-in amplifier. The polarization state of the laser is controlled using a half-wave plate (HWP) combined with a polarizer, and the laser is focused onto the sample using a 10× objective lens. (e) Laser position-dependent PTE characteristics: the nanohole array has higher resistivity than the electrode region, causing the dominant photocurrent direction to vary with the laser beam position.

To experimentally validate the narrowband photodetection by the plasmonic PTE effect, a 100 nm-thick hexagonal array of Au nanoholes was fabricated on a 200 nm-thick Si_3_N_4_ membrane (21584-10, TED PELLA INC.) (Figure 2a). Each nanohole has a diameter of 400 nm and a uniform lattice distance of 800 nm between neighboring nanoholes (Figure 2b). The size of the patterned region was 500 μm × 500 μm in the *x*–*y* direction, and Cu electrodes were precisely attached to the unpatterned strips on the Au film to minimize resistive noise during photocurrent measurement. Figure 2c shows the EOT spectrum of the fabricated hexagonal array of Au nanoholes (colored in red) compared with the calculated EOT spectrum, which was obtained using the FEM method to calculate Maxwell’s equations in the nanohole array. The simulation was performed using the same material parameters as the fabricated nanoholes, with a hole diameter of 400 nm, a period of 800 nm, and an Au film thickness of 100 nm (inset of Figure 2c). The optical transmission spectra of the fabricated sample were measured using a collimated halogen light source (FOK-150W, Fiber Optic Korea) in combination with a spectrometer (HR4000, Ocean Optics). The polarization of the incident light was aligned with the electrode direction using a linear polarizer. The experimental EOT spectrum closely matches the theoretical EOT spectrum, although slight discrepancies were observed due to geometric imperfections in the fabrication and the optical focusing effect of using a lens, as opposed to plane wave excitation in the simulation. From the theoretical and experimental EOT spectra, we confirmed that the hexagonal array of Au nanoholes provides sufficient SP resonance at a wavelength of 770 nm. In the PTE experimental setup, a tunable external cavity diode laser (ECDL, Toptica, DL pro) was focused on the hexagonal array of Au nanoholes with a spot size of ∼8.4 µm (1/*e* ^2^) using a 10× objective lens (Figure 2d). The laser polarization was controlled using a half-wave plate (HWP) combined with a linear polarizer. The photocurrent induced by the irradiated laser was measured with a lock-in amplifier (SR860, Stanford Research Systems) while the incident laser was modulated with a chopper system (SR540, Stanford Research Systems).

Based on the theoretical heat source distribution around the nanohole, as shown in Figure 1b, the photocurrent generated at each nanohole radiates outward from its center. The photocurrent produced at each hole can superimpose with photocurrents generated at neighboring nanoholes to form a collective flow. However, the flow of the generated photocurrent encounters varying resistance depending on the laser beam’s position within the pattern due to the increased sheet resistance in the nanohole array compared to a flat surface. This variation results in different photocurrent detection characteristics for identical laser power irradiation. Figure 2e presents the measured photocurrent as a function of the laser beam’s position along the electrode. When the laser beam passes over the electrode regions, the generated photocurrent is nearly zero, indicating that an unpatterned flat Au surface does not induce any PTE effect. When the laser beam fully illuminates the nanohole array (position 1), localized heat is efficiently generated from each nanohole due to SP resonance, producing a substantial photocurrent of −10 nA. However, as the laser beam moves toward the pattern’s center, the photocurrent gradually decreases. Due to the higher resistance of the nanohole array region compared to a flat Au surface, when the laser beam is focused on position 1, the left electrode region exhibits lower resistance than the nanohole array region on the right, resulting in less photocurrent loss to the left than to the right. According to this mechanism, the resistance encountered by the photocurrent varies as the laser beam position changes, determining the dominant direction of photocurrent flow. At the center of the nanohole array region (position 2), the generated photocurrent experiences nearly symmetrical resistance in both electrode directions, resulting in a photocurrent near zero. As the laser beam is further shifted to the right side of the patterned region (position 3), the photocurrent reappears with the opposite sign and nearly the same magnitude as observed at the left edge (position 1). The number of nanoholes within the illuminated area also affects heat generation, so the peak photocurrent occurs not at the edge of the nanohole region but where the laser spot fully covers the nanohole array. The effect of laser spot size on photocurrent magnitude is shown in Figure S1. We confirmed that the photocurrent generated by the Seebeck effect is influenced by the structural resistance within the device, which determines the direction of current flow. These findings allow us to identify the optimal laser beam position to achieve maximum photocurrent.

### 2.2 Optoelectronic performance of plasmonic PTE device

**Figure 3:**
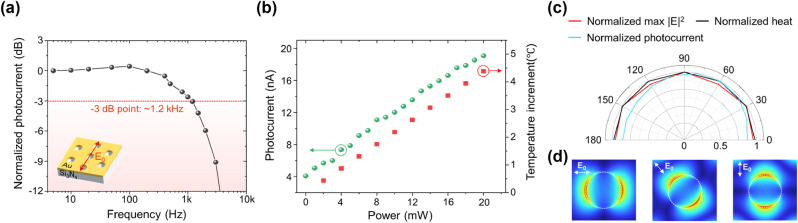
The optoelectronic characterization of the PTE device. (a) The measured photocurrent in the PTE device at different modulation frequencies using the optical chopper. (b) Laser power-dependent photocurrent and localized heat generation. The green circles represent the measured photocurrent from the PTE device, while the red squares correspond to the localized temperature increase generated in the Au nanohole array. (c) Polarization dependence of PTE generation, showing the measured photocurrent signal as the incident laser polarization, varies from 0° to 360° in 6° increments (sky blue line), alongside the calculated maximum intensity (red line) and localized heat (black line). (d) Calculated E-field distribution in the nanohole as a function of the polarization direction of the incident light.

To evaluate the optoelectronic performance of the PTE device, a 3 dB bandwidth measurement was conducted, along with an assessment of linearity in optical power measurement. In this measurement, the wavelength of the ECDL laser was set to 770 nm, with its polarization aligned parallel to the nanohole array (see inset of Figure 3a). The laser beam was fixed at position 1, as shown in Figure 2e, to achieve the maximum photocurrent signal. Figure 3a shows the frequency bandwidth characteristics of the PTE device. By increasing the rotation speed of the chopper connected to the lock-in amplifier, the input laser was gradually modulated from 1 Hz to 20 kHz. The average power of the modulated laser was set to be the same at all modulation frequencies. The 3 dB bandwidth of the PTE device was 1.2 kHz, indicating the optical detection bandwidth of the device. Additionally, the rise time of the PTE device was calculated using the relationship denoted as

(1) $$\tau_{r}≅\frac{0.35}{f_{-3dB}}$$

where *τ*_*r*_ is rise time and *f*_−3dB_ is the frequency at the −3dB point. According to this equation, response time of the PTE device is calculated to be approximately 0.292 ms. The response time is considerably faster than the typical response time of reported PTE-based photodetectors, which ranges from a few to several hundred milliseconds [37], [38], [39], [40]. Additionally, we evaluated the long-term stability of the photocurrent response to investigate the effect of accumulated heat during laser exposure (Supplementary Figure 3). The measurements were conducted under continuous exposure of a CW laser for 5 h. The average photocurrent measured during this period was 14.37 nA, with a standard deviation of 0.3055 nA. The calculated relative standard deviation (RSD) was 2.14 %, indicating stable performance throughout the entire measurement period. These results show that the Au plasmonic structure did not accumulate heat during the laser exposure. This confirms the device’s suitability for long-term operation in practical environments without degrading its detection capability.

Figure 3b shows the linearity of the photocurrent response to the laser power irradiated onto the Au nanohole array and the localized heat source generation as a function of laser power, calculated by FEM simulation. The ECDL laser, with a wavelength of 770 nm, was irradiated onto the PTE device, and the photocurrent signal was measured as the laser power was incrementally increased from 0 mW to 20 mW in steps of 1 mW. In the numerical heat source simulation, a continuous electromagnetic wave with a wavelength of 770 nm was modeled as a plane wave, and its power was varied from 2 mW to 20 mW in 2 mW increments. The measured photocurrent increased linearly with the incident laser power, consistent with theoretical results on localized heat generation. The calculated results show that the localized heat generated around the nanoholes increases linearly with the incident light energy. These findings indicate that the localized heat induced by SP resonance increases linearly with laser energy, leading to a corresponding linear increase in photocurrent generation according to the Seebeck effect. This linear response demonstrates that the plasmonic-PTE photodetector can quantitatively measure laser energy, with a responsivity of approximately 0.95 μA/W, as calculated from the experimental results. The device’s responsivity could be further enhanced by utilizing plasmonic structures that induce greater field intensity (see Supplementary Figure 4 for details).

In the square-array plasmonic EOT structure first introduced by Ebbersen [41], the resonance condition varies depending on the polarization of the incident light relative to the periodic direction of the holes. Consequently, different PTE effects are observed for each polarization state, requiring additional calibration to account for polarization under the same light energy, which can introduce errors in the measurement signal. To overcome these limitations, a hexagonal array of nanoholes was used in these experiments instead of the conventional square array. Figure 3c shows the polarization-dependent photocurrent measurement in the PTE device, with linear polarization swept from 0° to 180° in 30° increments. Interestingly, while the calculated electric field enhancement distribution in the nanostructure follows the rotating polarization state (Figure 3d), the measured photocurrents are nearly identical for all polarization states of the incident light. Note that due to the hexagonal geometry of the nanohole array, the maximum electric field enhancement is unaffected by the polarization state (red line in Figure 3c). Thus, a consistent amount of localized heat is generated due to steady electric field enhancement, although the heat source distribution rotates with the polarization angle. However, the experimental results confirm that this rotational characteristic of the heat source distribution does not significantly impact the measurable photocurrent. The minor variations observed in the measured photocurrent with changes in the polarization of the incident laser are attributed to slight shifts in the laser beam position on the PTE nanodevice, caused by minor changes in the optical path during the rotation of the half-wave plate and linear polarizer. These positional shifts, as discussed in Figure 2, can lead to variations in photocurrent even under the same laser power. These results experimentally validate the polarization independence of the hexagonal array of Au nanoholes that generates the plasmonic PTE effect in the device, confirming its potential as a photodetector for measuring light energy, regardless of incident light polarization.

### 2.3 Narrowband photocurrent generation and its tunability

**Figure 4:**
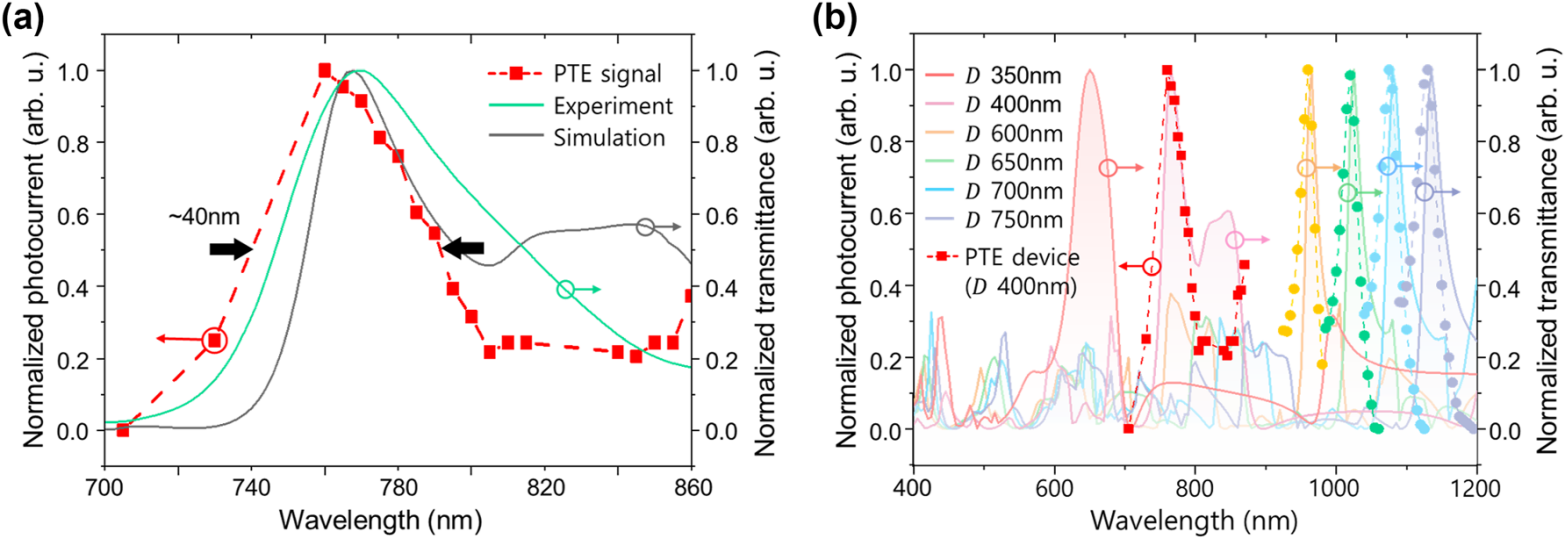
Spectral response of the PTE device. (a) Detection bandwidth of the PTE device (red square) compared with experimental (green curve) and theoretical (gray curve) transmittance spectra. The FWHM bandwidth of measured photocurrent is approximately 40 nm. (b) Numerically calculated transmittance spectra for nanohole diameters (*D*) from 350 nm to 750 nm, compared with the measured photocurrent response of the PTE device (red square). The field enhancements were overlaid on the EOT spectra as matching colored dots to align with the corresponding EOT spectrum. The pitch was set to 2*D*, forming a hexagonal array, with an Au thickness of 100 nm.

The main reason for using SP resonance for localized heat generation and photon-to-electron conversion is its strong wavelength dependence, which enables narrowband photodetection without requiring additional optical filtering. Figure 4a shows the measurement results of the narrowband photocurrent response of the PTE device. The incident laser wavelength was precisely scanned from 760 nm to 860 nm in 5 nm steps using the ECDL laser. Additional laser diodes with wavelengths of 700 nm (HL7001MG, Thorlabs) and 730 nm (HL7302MG, Thorlabs) were also used, as the ECDL laser does not cover the entire spectral range from 700 nm to 900 nm. The measured FWHM spectral bandwidth of the PTE device is approximately 40 nm, with the maximum photocurrent observed at a wavelength of 760 nm, showing a spectral peak difference of 10 nm from the peak optical transmission through the PTE device. This difference indicates that, while the resonant optical transmission can be influenced by other plasmonic longitudinal modes formed along the Au/Si_3_N_4_ interface, the PTE effect is attributed solely to lateral plasmonic resonance formed along the Au surface. This suggests that the spectral responses of the two physical phenomena behave similarly but are not induced by exactly the same surface plasmon resonance modes. Numerical calculations of the electric field enhancement on the Au nanohole show maximum enhancement at a wavelength of 760 nm, corresponding to the maximum photocurrent condition (see Supplementary Figure S2 for details). The FWHM bandwidth is about 40 nm, offering wavelength selectivity comparable to or even greater than that of the narrowband photodetectors listed in Table 1. Moreover, compared to other works, it has the capability of wavelength tuning across the visible and NIR regions, enabling more flexible wavelength adjustment over a broad spectral range.

**Table 1:** Summary of narrowband photodetectors.

Spectral range	Strategy	Material	Response peak (nm)	FWHM (nm)	Ref.
VIS	Narrowband absorber	BET/Zn_0.9_Mg_0.1_O	530	60	[42]
VIS	CCN	CH_3_NH_3_Pbl_2_Br	650	80	[13]
VIS	Nanowire	Si	400–650	50	[43]
NIR	CCN (EDN)	NT812:Y_6_	860	50	[11]
NIR	CCN	MAPbl_3_/CuSCN	810	95	[44]
NIR	Microcavity	ZnPc:C_60_	810–1,550	36	[45]
NIR	Nanograting	Si/PBDBT-DTBT:BTP-4F	895	26	[46]
			912	38	
			945	127	
			977	201	
NIR	CCN	PCDTBT:PC_70_BM	950	90	[47]
NIR	Plasmonic grating	Si	1,295–1,635	85	[48]
NIR	Tamm plasmon	PEDOT:PSS/Au	1,500	≈188	[49]
NIR	Tamm plasmon	Ge/Au	1,600–1,650	–	[50]
NIR	Plasmonic nanohole array	Au	760 (Exp.)	40	This work
VIS-NIR			650–1,150 (Sim.)	50 (VIS), 30 (NIR)	

CCN (charge collection narrowing), EDN (exciton dissociation narrowing).

An additional advantage of developing narrowband photodetectors based on plasmonic resonance is the ability to selectively tune the narrowband photocurrent response to a desired wavelength by adjusting the geometric design. Figure 4b shows the simulation results of the EOT spectrum calculated by adjusting the geometric structure of the nanostructure to tune the surface plasmon resonance conditions. While the SP resonance characteristics can be tuned by exploiting different materials or adjusting the film thickness, the nanohole periodicity was modified to achieve fine control over the resonance wavelength in these calculations. The resonance wavelength was controlled by adjusting the nanohole diameter *D* in a hexagonal array with a period of 2*D*, while maintaining an Au film thickness of 100 nm in all cases. The SP resonance conditions were analyzed through the calculated EOT spectrum. As the period 2*D* decreases, plasmon resonance occurs at shorter wavelengths, and as 2*D* increases, plasmon resonance shifts to longer wavelengths. As expected, the maximum field enhancement at each condition (indicated by dots) shows a slight mismatch with the EOT peak wavelength. However, the EOT spectrum provides solid experimental evidence for evaluating the designed PTE characteristics. This confirms that narrowband responses can be achieved in the visible range and the near-infrared range above 1,000 nm. The narrowband photocurrent response of the fabricated device closely matches the EOT spectrum predicted by the simulation, confirming that the resonance wavelength was accurately implemented as designed. These results demonstrate the potential for designing narrowband photodetectors with high sensitivity to specific wavelengths.

## 3 Conclusions

We developed a narrowband photodetector with high wavelength tunability by utilizing the PTE effect induced by SP resonance. By implementing a hexagonal array of Au nanoholes, we selectively enhanced electric field on the Au nanoholes at specific resonant wavelengths to detect its optical power. The plasmonic fields generate localized heat through a nonradiative decay process, which in turn induces photocurrent via the Seebeck effect. This approach enables a narrowband photocurrent response with high wavelength selectivity without requiring an external bias. Notably, the adoption of a hexagonal plasmonic structure, which efficiently generates heat and is insensitive to polarization, allows the device to achieve consistent light detection regardless of the incident light’s polarization state. The fabricated device demonstrated a linear responsivity of 0.95 μA/W and a narrow spectral response with a peak at 760 nm and an FWHM of 40 nm. This response closely matches the theoretical and experimental EOT spectra of the plasmonic structure, verifying that the resonant wavelength of the device was precisely implemented as designed. These results demonstrate that the detection wavelength can be freely tuned through geometric design of the plasmonic structure, enabling narrowband response characteristics across various wavelength ranges while QD-based systems offer excellent optical properties, they are often limited by the complexity of controlling their properties during chemical synthesis and the challenges in achieving uniform deposition on a sample surface. The plasmonic photodetector proposed in this study utilizes well-defined nanostructures on metallic surfaces. This approach enables high reproducibility, tunable optical properties, and scalable fabrication through methods like nanoimprint lithography (NIL). Moreover, the device achieves narrowband photoresponse without external bias voltage, significantly reducing operational complexity, electrical noise, and power consumption. These features make it a practical and cost-effective alternative to QD-based systems and conventional photodetectors. Consequently, the device developed in this study offers high applicability in diverse fields, including optical communications, sensing, and precise spectral analysis.

## Supplementary Material

Supplementary Material Details
