# Supplementary material for: Polarization-independent narrowband photodetection with plasmon-induced thermoelectric effect in a hexagonal array of Au nanoholes
Source: Nanophotonics. 2025 Feb 17;14(10):1615–24. doi: 10.1515/nanoph-2024-0643 (PMC12116267; doi:10.1515/nanoph-2024-0643)
Supplement: Supplementary file 1 — Supplementary Material Details [file j_nanoph-2024-0643_suppl_001.docx]

**Supplementary Material**

**Polarization-independent narrowband photodetection with plasmon-induced thermoelectric effect in a hexagonal array of Au nanoholes**

Sehyeon Kim^1, †^, San Kim^1, †^, Jae-young Kim^1^, Tae-In Jeong^1^, Munki Song^1^, Seungchul Kim^1,2^*

† Sehyeon Kim and San Kim contributed equally to this work.

^1^ Department of Cogno-Mechatronics Engineering, College of Nanoscience and Nanotechnology, Pusan National University, Busan 46241, Republic of Korea
^2^ Department of Optics and Mechatronics Engineering, College of Nanoscience and Nanotechnology, Pusan National University, Busan, 46241, Republic of Korea

Corresponding Author: Seungchul Kim (s.kim@pusan.ac.kr)

**Keywords**: surface plasmon resonance; narrowband photodetection; photothermoelectric;


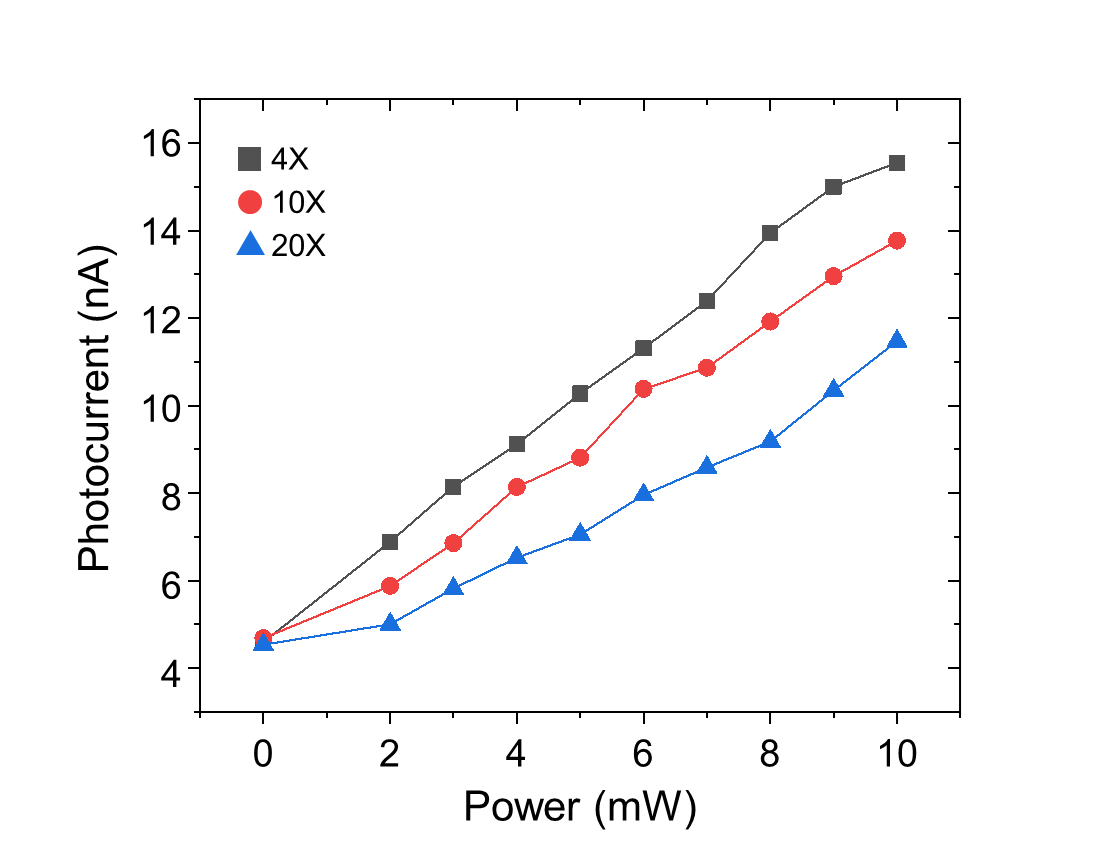


**Supplementary Figure 1.** Power dependence of photocurrent based on the magnification of the focusing objective lens. The laser beam spot sizes are approximately 14.6 µm at 4X magnification, 8.4 µm at 10X magnification, and 4.2 µm at 20X magnification.


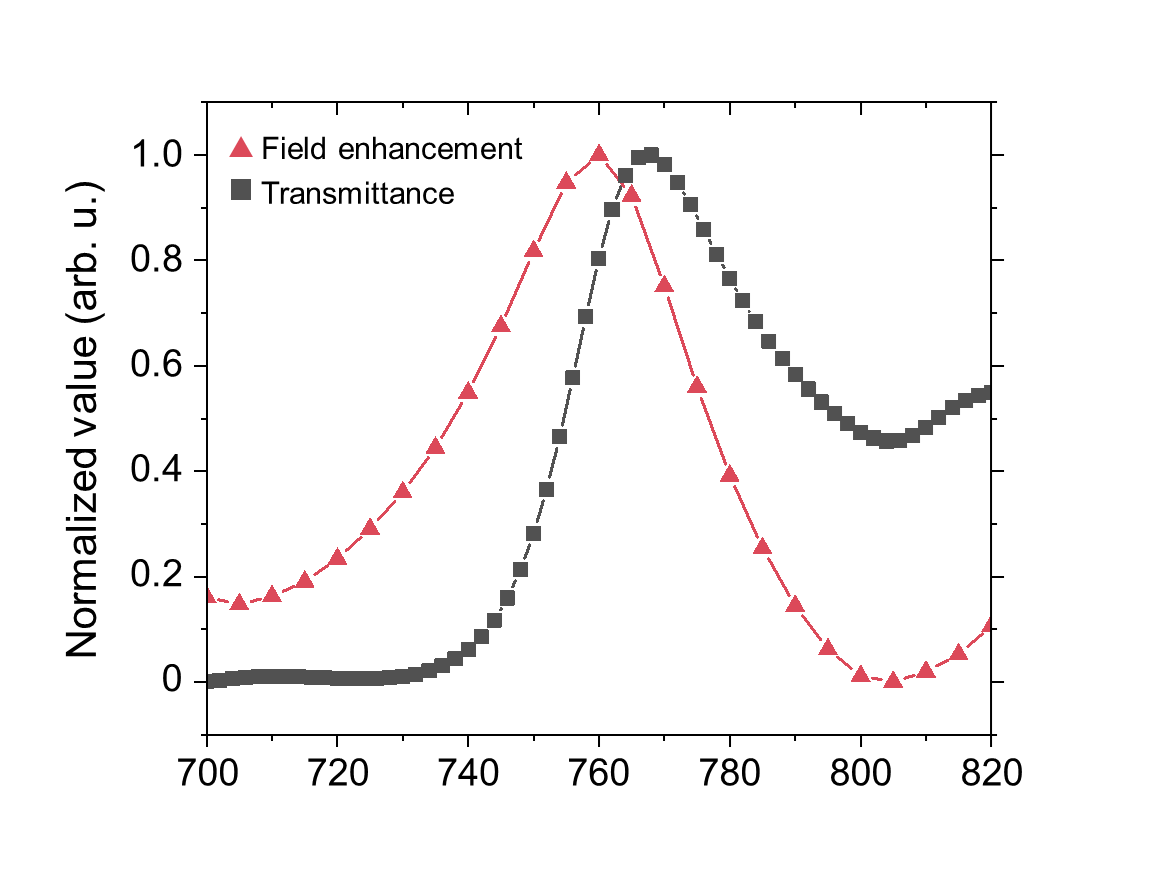


**Supplementary Figure 2.** Numerically calculated transmittance and field enhancement of the Au nanohole array (D = 400 nm).

**
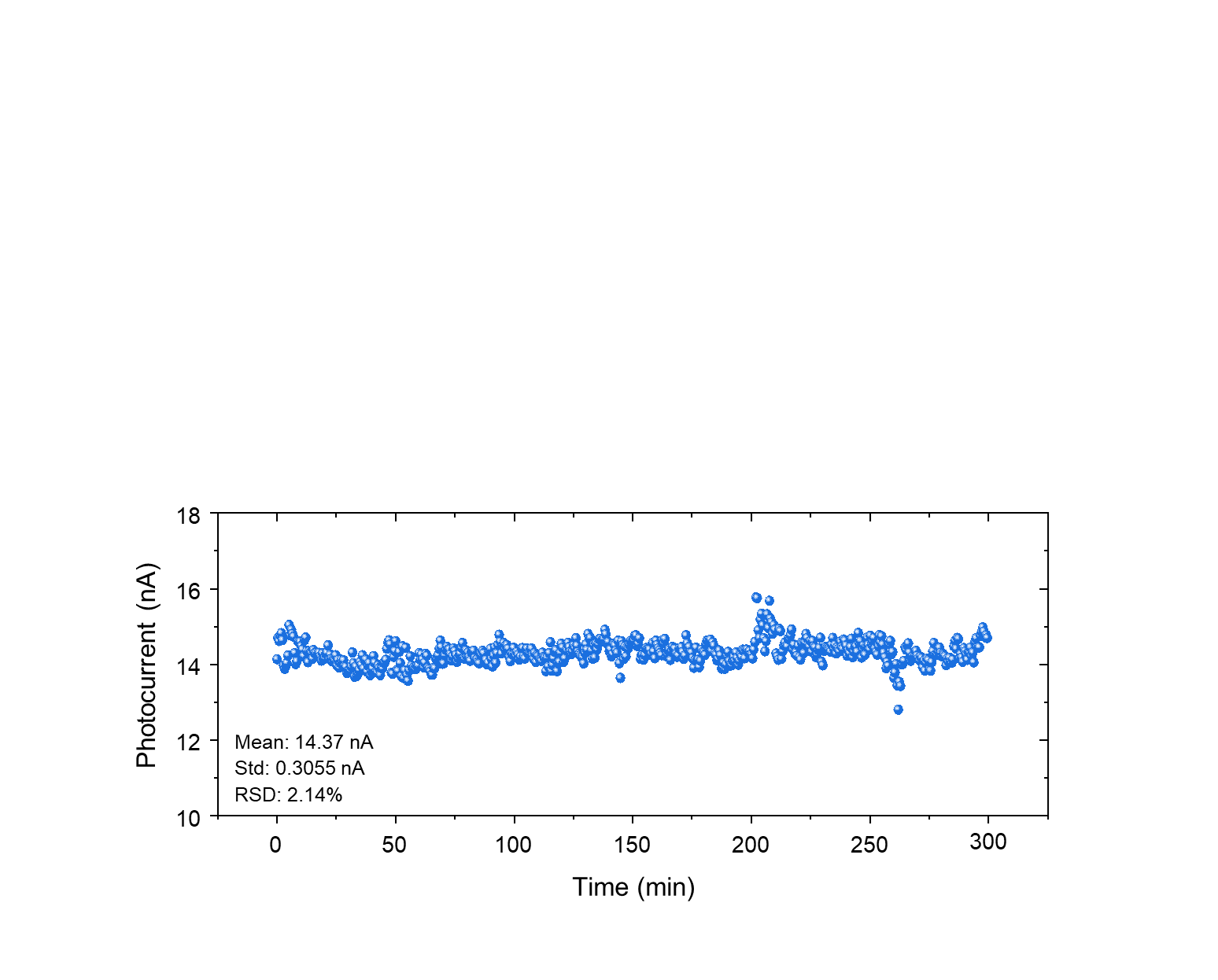
**

**Supplementary Figure 3.** Long-term stability of the generated photocurrent over time. Measured under continuous laser illumination with a power of 10 mW at ambient temperature (24 °C) for 5 hours.


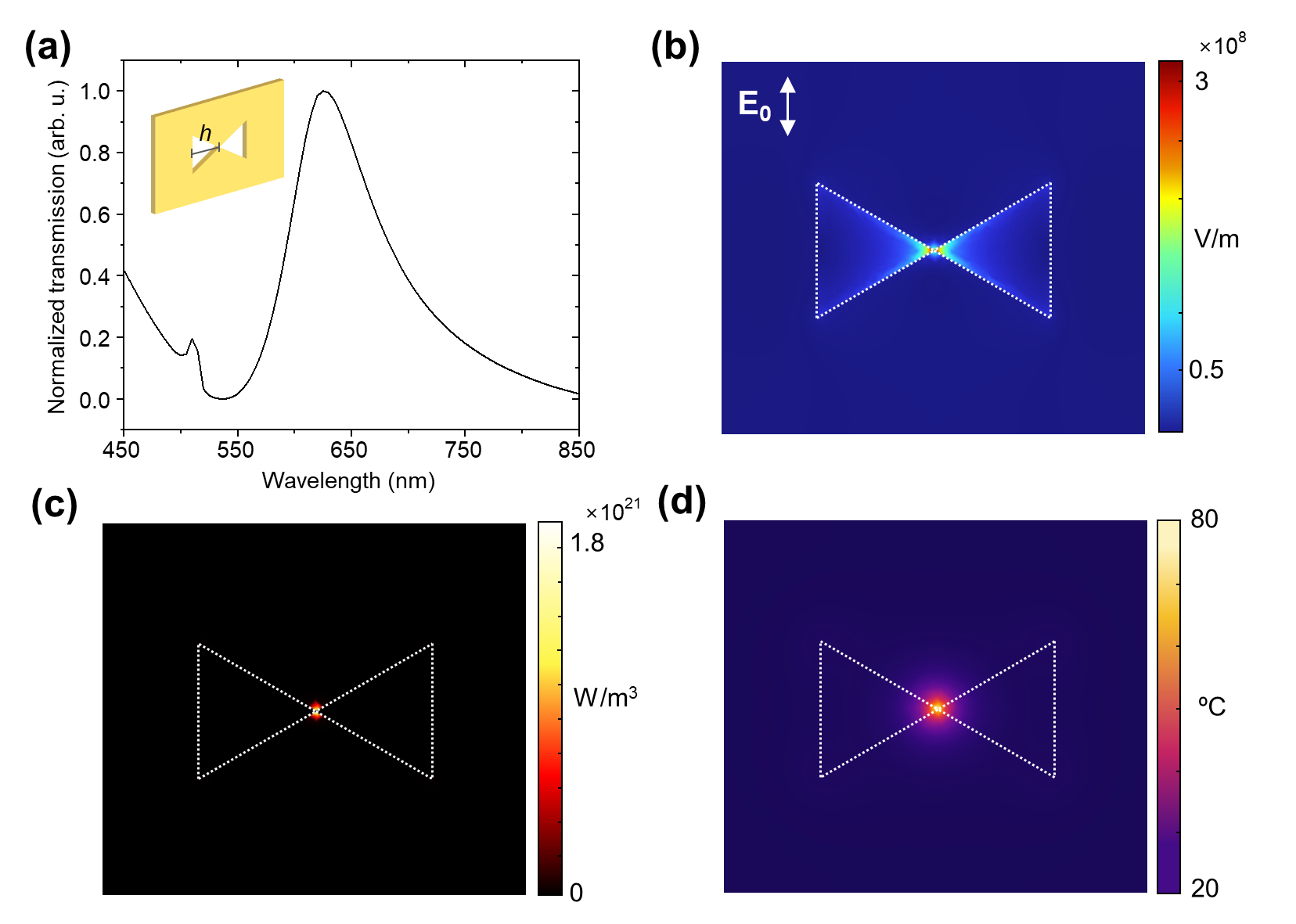


**Supplementary Figure 4**. (a) Calculated transmission spectrum of a bow-tie nanohole structure. Note that there is no gap between the two Au triangle shapes, each with a height (*h*) of 90 nm, and the gold film has a thickness of 50 nm. (b) Calculated electric field distribution, (c) calculated heat source density, and (d) heat distribution under a 10 mW laser with a wavelength of 620 nm. The temperature increases by approximately 60 °C, likely due to the increased field intensity and the highly concentrated heat source density at specific points in the structure.
